# Supplementary material for: Drivers of ‘voluntary’ recruitment and challenges for families with adolescents engaged with armed groups: Qualitative insights from Central African Republic and Democratic Republic of the Congo
Source: PLOS Glob Public Health. 2023 May 24;3(5):e0001265. doi: 10.1371/journal.pgph.0001265 (PMC10208483; doi:10.1371/journal.pgph.0001265)
Supplement: S1 Appendix — Guide A. Individual Interviews for Boy/Girl Children and Adolescents Life Line Tool: Previously engaged with armed groups. Guide B. Individual interviews for caregivers of children and adolescents previously engaged with armed groups. Guide C. Individual interviews for caregivers of children and adolescents previously engaged with armed groups. (DOCX) [file pgph.0001265.s001.docx]

**S1 Appendix: Data collection instruments**

#### Guide A. Individual Interviews for Boy/Girl Children and Adolescents Life Line Tool: Previously engaged with armed groups

Hi, my name is [NAME] and I am excited to talk to you.

1. First, I would like to learn a little more about you and your daily life. Can you tell me about a typical day in your life? What do you do?
   1. What do you do in the morning, afternoon and evening?
   2. What do you like doing best? What are they and why?
   3. Who do you spend time with during the day?
      1. Whom do you live with and where?
   4. Are you working? What kind of work?
2. I am going to ask you some general questions to understand your general details. We ask all persons we are interviewing these questions to help us understand the profile of the people we have spoken with.
   1. How old are you?
   2. Can I confirm that you live here in _____ village/town/camp
   3. Sex: Male/Female/ Other (circle one)
   4. Have you ever been displaced due to conflict: Yes/No (circle one)
   5. Partnered/married: Yes/No (circle one)
   6. Number of years living in current location:
   7. Family members currently living with (list all):
   8. Do you have children? If so, how many?
      1. What is the age of your oldest child?
      2. What is the age of your youngest child?
      3. What gives you most joy about your child/ren?
      4. What major challenges do you have in supporting your child/ren?
3. In order to be able to have good information, we need to ask people six direct questions about whether they have a certain type of disability and how much difficulty they have from it. We will use this information to get a better sense of the needs among older persons living here.

|  | No - no difficulty | Yes – some difficulty | Yes – a lot of difficulty | Cannot do at all |
| --- | --- | --- | --- | --- |
| 1. Do you have difficulty seeing, even if wearing glasses? |  |  |  |  |
| 2. Do you have difficulty hearing? Do you use a hearing aid? |  |  |  |  |
| 3. Do you have difficulty walking or climbing steps? |  |  |  |  |
| 4. Do you have difficulty remembering or concentrating? |  |  |  |  |
| 5. Do you have difficulty (with self-care such as) washing all over or dressing? |  |  |  |  |
| 6. Do you have difficulty communicating, for example understanding or being understood? |  |  |  |  |

- 1. Do you know if you have had your difficulty since birth, or did it start later? If later at what age was that?

**Now, I am going to ask you questions specific to the topic of child recruitment in armed groups. All the questions have no right or wrong answer, therefore you can express your answers in any way that you are comfortable with. It is important for us to understand your perspective.**

**I want to draw out a timeline of your life to better understand positive or negative experiences you may have had. Imagine that this line on this paper represents your life. On this side, you are younger and it is before you were involved with the armed groups. I want to write out what your life was like during this time. I will write the happy experiences on the top and the times when you were sad or upset underneath the line. Does this make sense?**

1. Great, can you tell me what your life was like before you joined or were engaged or in contact with the armed group?
   1. Relationship with family: Tell me about your relationship with your family.
      1. How was your relationship with your parents?
      2. How different was this relationship when you were less than 10 years old and between 10 years old and now?
      3. How was your relationship with your brothers and sisters?
   2. Relationship with friends: Tell me about your relationships with your friends.
      1. With whom did you spend time and how do you spend your time?
      2. What made these relationships good relationships?
      3. What did you not like about relationships with friends?
   3. Education: Did you go to school?
      1. If yes, did you like it? Why?
      2. If you stopped going to school, why did you stop?
         1. When did you stop?
   4. Economic situation: How did your family earn money or a living?
      1. How did you earn money?
      2. What was your economic situation? Could you afford everything that you needed? Tell me about this.
   5. Tell me about your good / happy experiences?
   6. Tell me about your bad / sad experiences?
2. What was happening in your life that made you join the armed group?
   1. Economic reasons: Did you think about earning money when you wanted to join the armed group? Why or why not?
   2. Belief in cause: Did you believe in the cause of the armed group? Why or why not?
   3. Household/parental support: Did your parents know that you were going to be engaged with the armed groups?
      1. If they knew, did they want you to join? Why or why not?
   4. Social support: Did you know anyone else who was joining? Tell me about them.
      1. Did you think it is good? Why or why not?
      2. Did you have friends who joined? Tell me about this.
   5. What age were you when you joined / became involved? What age when you got out?
3. What were some of the fears or thoughts you might have heard before joining the armed groups?
   1. How did you manage these fears?
4. What did your parents/caregivers think about you joining?
   1. Were they happy, sad, upset?
   2. Did they want you to join? Why or why not?
   3. Do they like the armed groups? Why or why not?

Thank you so much for sharing that. Now this period on the line represents the time when you were with the armed group and fighting.

1. What you’re your typical day like when you were in the group?
   1. What did you do in the mornings? In the afternoons? In the evenings?
   2. Did you live with the group? Tell me about this.
   3. Did you go home at all when you were with the armed group? What did you do when you went home?
      1. What were some of the things you liked about going home?
      2. What was your role in your family at that time?
2. What did your family think about you joining the armed group?
   1. What were your parents’ reactions, were they sad, encouraging, or angry? Why?
3. Can you describe some of the experiences you had during your time with the group?
   1. I know this can be difficult, so maybe you can think of one time when you may have feel happy or hopeful to start?
   2. Economic advantages: Did you receive an income from the group? Did they give you other things such as clothes or food when you were part of the group? Tell me about this.
   3. Social support: Tell me about your social networks in the group. Did you have friends? Did you have support or mentors? Tell me more about this subject.
   4. Thank you for sharing. Now were there any bad experiences that you would like to share?
      1. Why?
      2. What made you afraid? What did not make you afraid?
4. How long did you stay with the armed group? Why did you decide to stay with them?

Thank you – I know it can be hard to think about the past. Now this part on the line represents where you are now in life, back with your family and community.

1. First, tell me what joys or fears you had when you were planning to return home. Why?
2. Can you describe some positive things that have happened in your life now that you have returned? I will write them above the line.
3. Relationship with family: Tell me about your relationship with your family. How is your relationship with your parents? Has this relationship changed from before? How?
   1. Is the relationship with your mother different from the relationship with your father? How?
   2. What do you like about the relationships? What do you wish was different?
4. How was your relationship with your brothers and sisters? Have these relationships changed from before? How?
   1. With your siblings, is there any difference in these relationships with your brothers and sisters? (Is it different for the boys or the girls?)
5. Relationship with friends: Tell me about your relationships with your friends. Have these relationships changed from before? How?
   1. Is there any difference in relationships between your male or female friends?
   2. What is the perception of your friends since you returned from the armed group? What is positive and what is negative?
6. Education: Do you currently go to school?
   1. If yes:
      1. What are some of the fears you had before joining school? Did these fears come true?
      2. What do you like about school?
      3. What don’t you like? What do you wish was different?
   2. If no: Why not?
      1. What is the main barrier to you going back to school?
      2. How does this make you feel?
7. Economic situation: How do you make a living or earn money now? Do you receive support from your family? Tell me more about this subject.
8. Thank you, can you also describe some of the challenges you have had since you returned? I will write them below the line.
   1. Economic difficulties?
   2. What is your relationship with others in the community?
   3. Do you experience stigmatization in the community, from your family or your friends?
      1. How do you feel?
   4. Do you have disagreements with your family? Your spouse (if married)?
9. How have COVID-19 and the lockdowns changed the relationships in your family? Between you and your parents? Between you and your siblings? Between you and your spouse (if married)?
   1. How has COVID-19 and the lockdowns influenced your overall wellbeing?

Now finally, we are to the end of the line. This represents your hopes for the future. Could you share with me what you want your future to look like?

1. What are some positive things you are looking forward to? What do you want for yourself?
   1. Thinking about your life in the next 5 years, what would you like to be doing between now and then?
      1. What would you like to have between now and then?
   2. Thinking about your life in 10 years, what would you like to be doing between now and then?
      1. What would you like to have in your life at that point?
2. What are some things that you want with your family and relationships? Why?
   1. How do you want to provide for your needs? What do you want to do for work?
      1. What will make it possible for you to achieve this?
      2. What barriers will make it difficult to achieve this?
   2. What do you want / need in the future to support your family relationships?
      1. What will make it possible for you to achieve this?
      2. What barriers will make it difficult to achieve this?
3. What worries you about the future?
4. What gives you hope about the future?
5. Sometimes we think about the future we want and we think the ideas are too big or not possible. No answer is wrong, no matter how big or small your ideas are. How would you like to contribute to this future that you imagine for yourself, even if the idea is big or small? What can help you achieve that future that you imagine?
6. Is there anything else you would like me to know about?

Thank you so much for all of your help today. We greatly appreciate it. If you would like to talk more about how you are feeling after this interview, your caseworker is available and you can talk to them.

#### Guide B. Individual Interviews for Boy/Girl Children and Adolescents Life Line Tool: At-risk adolescents (not engaged with armed groups)

Hi, my name is [NAME] and I am excited to talk to you.

1. First, I would like to learn a little more about you and your daily life. Can you tell me about a typical day in your life? What do you do?
   1. What do you do in the morning, afternoon and evening?
   2. What do you like doing best? What are they and why?
   3. Who do you spend time with during the day?
      1. Whom do you live with and where?
   4. Are you working? What kind of work?
2. I am going to ask you some general questions to understand your general details. We ask all persons we are interviewing these questions to help us understand the profile of the people we have spoken with.
   1. How old are you?
   2. Can I confirm that you live here in _____ village/town/camp
   3. Sex: Male/Female/ Other (circle one)
   4. Have you ever been displaced due to conflict: Yes/No (circle one)
   5. Partnered/married: Yes/No (circle one)
   6. Number of years living in current location:
   7. Family members currently living with (list all):
   8. Do you have children? If so, how many?
      1. What is the age of your oldest child?
      2. What is the age of your youngest child?
3. In order to be able to have good information, we need to ask people six direct questions about whether they have a certain type of difficulty and how much difficulty they have from it. We will use this information to get a better sense of the needs among older persons living here.

|  | No - no difficulty | Yes – some difficulty | Yes – a lot of difficulty | Cannot do at all |
| --- | --- | --- | --- | --- |
| 1. Do you have difficulty seeing, even if wearing glasses? |  |  |  |  |
| 2. Do you have difficulty hearing? Do you use a hearing aid? |  |  |  |  |
| 3. Do you have difficulty walking or climbing steps? |  |  |  |  |
| 4. Do you have difficulty remembering or concentrating? |  |  |  |  |
| 5. Do you have difficulty (with self-care such as) washing all over or dressing? |  |  |  |  |
| 6. Do you have difficulty communicating, for example understanding or being understood? |  |  |  |  |

- 1. Do you know if you have had your difficulty since birth, or did it start later? If later at what age was that?

**Now, I am going to ask you questions specific to the topic of child recruitment in armed groups. All the questions have no right or wrong answer, therefore you can express your answers in any way that you are comfortable with. It is important for us to understand your perspective.**

**I want to draw out a timeline of your life to better understand positive or negative experiences you may have had. Imagine that this line on this paper represents your life. On this side, you are younger. I want to write out what your life was like during this time. I will write the happy experiences on the top and the times when you were sad or upset underneath the line. Does this make sense?**

1. Great, can you tell me what your life is like now?
   1. Relationship with family: Tell me about your relationship with your family.
      1. How is your relationship with your parents?
      2. How different was this relationship when you were less than 10 years old and between 10 years old and now?
      3. How is your relationship with your brothers and sisters?
   2. Relationship with friends: Tell me about your relationships with your friends.
      1. With whom do you spend time and how do you spend your time?
      2. What makes these relationships good relationships?
      3. What do you not like about relationships with friends?
   3. Education: Do you go to school now, or did you go in the past?
      1. If yes, did you like it? Why?
      2. If you stopped going to school, why did you stop?
         1. When did you stop?
   4. Economic situation: How does your family earn money?
      1. How do you earn money?
      2. What is your economic situation? Can you afford everything that you need? Tell me about this.
   5. Tell me about your good / happy experiences?
   6. Tell me about your bad / sad experiences?
2. Now during this time, there have been many children in your community who were joining armed groups. Did you ever think about joining? Why or why not?
   1. Economic reasons: Did you think about earning money when you wanted to join the armed group? Why or why not?
   2. Belief in cause: Did you believe in the cause of the armed group? Why or why not?
   3. Household/parental support: Did your parents want you to join? Why or why not?
      1. Do they like the armed groups? Why or why not?
   4. Social support: Did you know anyone else who was joining? Tell me about them.
      1. What did you think about the other kids who were joining? Did you think it is good? Why or why not?
      2. Did you have friends who joined? Tell me about this.
3. Why did you decide to not join the armed groups? Tell me more about this.
4. What did your caregivers/parents say about you not joining?
   1. Were they happy, sad, upset?

Thank you for sharing with me all of this information. I would like to know about your life now.

1. How have COVID-19 and the lockdowns changed the relationships in your family? Between you and your parents? Between you and your siblings? Between you and your spouse (if married)?
   1. How has COVID-19 and the lockdowns influenced your overall wellbeing?

Now finally, we are to the end of the line. This represents your hopes for the future. Could you share with me what you want your future to look like?

1. What are some positive things you are looking forward to? What do you want for yourself?
   1. Thinking about your life in the next 5 years, what would you like to be doing between now and then?
      1. What would you like to have between now and then?
   2. Thinking about your life in 10 years, what would you like to be doing between now and then?
      1. What would you like to have in your life at that point?
2. What are some things that you want with your family and relationships? Why?
   1. How do you want to provide for your needs? What do you want to do for work?
      1. What will make it possible for you to achieve this?
      2. What barriers will make it difficult to achieve this?
   2. What do you want / need in the future to support your family relationships?
      1. What will make it possible for you to achieve this?
      2. What barriers will make it difficult to achieve this?
3. What worries you about the future?
4. What gives you hope about the future?
5. Sometimes we think about the future we want and we think the ideas are too big or not possible. No answer is wrong, no matter how big or small your ideas are. How would you like to contribute to this future that you imagine for yourself, even if the idea is big or small? What can help you achieve that future that you imagine?
6. Is there anything else you would like me to know about?

Thank you so much for all of your help today. We greatly appreciate it. If you would like to talk more about how you are feeling after this interview, your caseworker is available and you can talk to them.

#### Guide C. Individual interviews for caregivers of children and adolescents previously engaged with armed groups

Hi, my name is [NAME] and I am very happy to talk to you today. As you know, we want to learn more about the experiences of children who were involved with armed groups like [NAME 2-3 armed groups as an example] and to learn how we can better support caregivers, like you, to help them be happy now and reduce risk of returning to the armed group in the future. Are you ready to begin the interview?

1. First, I would like to learn a little more about you and your daily life. Can you tell me about a typical day in your life? What do you do?
   1. Who do you spend time with during the day?
      1. Whom do you live with and where?
   2. Do you have children? How many? Hold old are they?
2. I am going to ask you some general questions to understand your general details. We ask all persons we are interviewing these questions to help us understand the profile of the people we have spoken with.
3. How old are you?
4. Can I confirm that you live here in _____ village/town/camp
5. Sex: Male/Female/ Other (circle one)
6. Have you ever been displaced due to conflict: Yes/No (circle one)
7. Partnered/married: Yes/No (circle one)
8. Number of years living in current location:
9. Family members currently living with (list all):
10. Do you have children? If so, how many?
    1. What is the age of your oldest child?
    2. What is the age of your youngest child?
11. Are you working? What kind of work?
12. Washington Group Questions

In order to be able to have good information, we are asking everyone we speak with six direct questions about difficulties they may have in performing some basic activities. We will use this information to get a better sense of the needs among persons living here.

|  | No - no difficulty | Yes – some difficulty | Yes – a lot of difficulty | Cannot do at all |
| --- | --- | --- | --- | --- |
| 1. Do you have difficulty seeing, even if wearing glasses? |  |  |  |  |
| 2. Do you have difficulty hearing? Do you use a hearing aid? |  |  |  |  |
| 3. Do you have difficulty walking or climbing steps? |  |  |  |  |
| 4. Do you have difficulty remembering or concentrating? |  |  |  |  |
| 5. Do you have difficulty (with self-care such as) washing all over or dressing? |  |  |  |  |
| 6. Do you have difficulty communicating, for example understanding or being understood? |  |  |  |  |

1. Do you know if you have had your difficulty since birth, or did it start later? If later at what age was that?

**I am now going to ask you questions specific to your child on the topic of child recruitment. All the questions have no right or wrong answer, therefore you can express your responses in a manner that you are comfortable with. It is important for us to understand your perception and your thoughts and opinions are very valuable.**

1. We understand that your son/daughter was engaged with armed groups.
   1. Could you share with me what their involvement was to your knowledge? (NOTE: Their involvement can include selling, carrying goods, washing clothes, cooking, and other tasks, not only active fighting).
   2. How do you feel about this? Were you proud, sad, upset, relieved or anything else? Why?
      1. Did you know the child was going to join the group? Did you speak with them about it before they left? Tell me more about this time.
   3. What is your opinion on the armed groups? Why?
   4. *(Note: ask this question based on the response in question 2a). If the child only had contact with the armed groups through a particular activity (e.g. selling), but was not working with them on a day to day basis :*
      1. Did the child only engage in this work/activity with the group or also with others in the community/market? How long was this contact?
      2. How did they get connected to the armed group?
      3. Do you worry about the child having any contact with the armed groups, and why?
2. We would like to understand more about what your son/daughter did while they were with the armed group.
   1. How old was your child when they were engaged with the armed group/became involved? How old when they got out / stopped?
   2. What is the reason that pushed/motivated them to get involved initially?
      1. Of the reasons you have mentioned, how much was this influenced by the economic situation of the family? Why or why not?
         1. What was your economic situation at this time? Did this influence them to join the group?
         2. Did your child think that they would receive an income from the group? And if yes, did they receive an income or another form of payment when they were in the group? Did they send money home?
      2. How much of this decision was influenced by political reasons or belief in the cause of the armed group? Why or why not?
      3. How much of this decision was influenced by parental / family support? Tell me about this.
         1. Would this decision to join and the family reaction have been different if the child was a boy/girl? How?
      4. How much of this decision was influenced by social reasons, for example their friends?
   3. Have they gone back to the armed groups since coming home? If yes, how often? What led to this happening?
3. What types of experiences did your child have while they were with the armed group?
   1. Tell me more about the experiences he/she had that might have been good? Why do you classify these as good experiences?
   2. Tell me more about his/her bad experiences? Why do you classify these as bad experiences?
   3. Have you talked about your child’s experiences with them? Why or why not?
      1. If you discussed it with them, what did you focus your discussion on?
      2. How did you do this? How often did you have this discussion with them?
      3. What did you wish you would have discussed but didn’t manage to discuss with them?
      4. What prevented you from discussing these with them?
   4. How do you think this experience has changed your child in terms of how they feel?
   5. Other than talking to your child, what else have you done to help your child feel more welcome at home?
      1. How do you know that this has helped?
4. We understand that now your child is back living with you. What has it been like to have them back in the home? (Alternative: we understand that your child is still with the armed groups. What does this mean for your family and your child’s siblings?)
   1. When you first saw your child when s/he returned, what was your reaction? Did you know they were coming back home that day?
      1. What did you do to make your child feel welcome?
   2. What is your relationship with your son/daughter like now?
   3. How has it changed from before they joined the armed group?
   4. Would this relationship have been different if your child was male/female?
   5. How would you describe the relationship? Is your child loving, happy, stressed, angry?
5. What are the relationships like between siblings family members and the child now?
   1. How did the relationships change from before?
      1. Would this relationship have been different if the child had been male/female?
      2. How would you describe the relationships? Are they loving, happy, stressed, angry?
   2. What are the relationships like between the child and other extended family members?
6. How did the relationships change from before?
7. How would you describe the relationships? Are they loving, happy, stressed, angry?
   1. Are the relationships different between male/female members of the family?
8. I would like to know more about the difficulties or challenges your child may be having now that they are back at home.
   1. What are some of the challenges your child is facing within the family?
   2. What are some of the challenges your child is facing in the neighborhood/community?
   3. Is your child facing any other challenges? Tell me more about this.
   4. How did you become aware of these challenges?
9. What are some of the challenges you are experiencing as a caregiver?
10. We want to better understand how best to support caregivers like yourself. Could you share with me some of your experiences related to the ongoing conflict?
    1. What experiences did you or your family have when your child was away/engaged with armed groups?
       1. Were you or any members of your family ever jailed, fined or punished because of your child’s involvement in the armed group? If so, tell me about what happened.
    2. How has your child’s time with the armed group influenced your overall wellbeing?
       1. Mental health, stress: Did it increase your stress? How? Why?
       2. Economic instability: Did it change the economic situation of your family? How? Why?
       3. Did it affect your relationship with other family members/relatives or with your neighbors?
    3. Do you think this has changed your role as a parent?
    4. (If married / living with a partner) How is your relationship with your spouse?
       1. Did your relationships with your partner change when your child was engaged with the armed group, whether positive or negative? How did it change? tell me more about this.
       2. Has your relationship changed since your child came home, whether positive or negative? How has it changed?
       3. Would your relationship with your partner have been any different if the child engaged with armed groups had been male/female?
11. How have COVID-19 and the lockdowns changed the relationships in your family? Between you and your child? Between you and your spouse?
    1. How has COVID-19 and the lockdowns influenced your overall wellbeing?

We want to develop a program for parents to help them support their children and families.

1. What skills or opportunities do you want to gain for helping you and your child right now, based on their experience with armed groups? This will help us understand what parents like you need help with.
   1. What things do you want to know how to do as a parent in order to better support your child?
   2. What competencies for raising your children would you like to learn?
   3. Thinking about the challenges you mentioned that you faced in supporting your child when they returned, what do you think IRC can do to support parents manage such challenges?
   4. What could have helped you personally to better support your child to feel welcome and do well in the home?
      1. How can IRC support parents like you in this process?
   5. What could have best helped you personally to better support your child manage the challenges they face once they returned to the community?
      1. How can IRC support parents like you in this process?
   6. What are the topics that you and other parents would want to speak about to help you better support your children?
   7. Or other types of support for your family?
2. If we were to develop a program that offered you skills and knowledge, such as a training session, how would you like it to be?
   1. Face-to-face or through other means (such as by phone)?
   2. What times of day?
   3. What would motivate you to participate?
   4. If you have a partner/spouse, how should we engage both of you in this program?
   5. How should we engage your children in such a program?
   6. Who else in the community should we work with?
3. Is there anything else we should know about how to best support caregivers and children returning from fighting forces?

Thank you so much for all of your help today. We greatly appreciate it.
