# Supplementary material for: Drivers of ‘voluntary’ recruitment and challenges for families with adolescents engaged with armed groups: Qualitative insights from Central African Republic and Democratic Republic of the Congo
Source: PLOS Glob Public Health. 2023 May 24;3(5):e0001265. doi: 10.1371/journal.pgph.0001265 (PMC10208483; doi:10.1371/journal.pgph.0001265)
Supplement: S2 Appendix — Table A. Key findings for adolescents by country: Risk and protective factors for engaging with armed groups and reintegrating with communities before, during, and after conflict. Table B. Key findings for caregivers by country: Risk and protective factors for adolescents engaging with armed groups and reintegrating with communities before, during, and after conflict. (DOCX) [file pgph.0001265.s002.docx]

**S2 Appendix: Overview of key findings by country**

**Table A. Key findings for adolescents by country: Risk and protective factors for engaging with armed groups and reintegrating with communities before, during, and after conflict**

| **Time period** | **Central African Republic** | **Democratic Republic of Congo** |
| --- | --- | --- |
| Pre-conflict | **Risk factors for engaging with armed groups**   - Belief in / affiliation with cause of the armed group - Poor economic conditions - Social influence of the community and caregivers   - Influence of community / friends mentioned more among boys - Parental support for engagement for economic reasons (e.g. income for the family), mentioned more among girls   **Protective factors for not engaging with armed groups**   - Positive social influence (e.g. positive relationships with family, friends not in the armed group) - Caregivers discouraged them from joining (reported by boys who did not engage with groups) | **Risk factors for engaging with armed groups**   - Poor economic conditions and the negative influence of this on family dynamics - Parental support for engagement for economic reasons (e.g. income for the family)   **Protective factors for not engaging with armed groups**   - Positive social influence (e.g. close-knit family dynamic and desire to support family economically and emotionally - Caregivers or other family members discouraged them from joining (reported by girls and boys who did not engage with groups, more often by boys) |
| Conflict | **Motivations to leave the armed group**   - Peer violence within the armed group - Poor living conditions - Frequent contact with families and/or living with family, who encouraged them to leave the group   - Caregiver disapproval of engagement   - Responsibilities at home (older adolescents)   **Motivations to stay with the armed group**   - Positive social dynamics within the group for some boys and girls:   - Emotional support from other youth   - Mentorship and protection from older youth and leaders - Some economic support | **Motivations to leave the armed group**   - Peer violence within the armed group, sexual and economic violence for girls, in particular - Poor living conditions - Limited ability to contact families while with group, and little social support within group - For those few who were in contact with families:   - Caregiver disapproval of engagement - Responsibilities at home (older adolescents)   **Motivations to stay with the armed group**   - Negative situation with family at home (no reason to return) |
| After the conflict | **Barriers to reintegration**   - No access to basic needs or livelihoods   - Desire to obtain a successful livelihood, but concern that conflict will persist and prevent community from developing - Social stigmatization from community and family members of all genders, mostly reported by girls (related to reputation of “being with” armed men) - Tension with family, and inability to discuss what happened to them with their family members | **Barriers to reintegration**   - No access to basic needs or livelihoods (e.g. previous source of income was through association with armed group) - Caregivers unable to provide for needs - Social stigmatization from family members, mostly reported by girls experiencing stigma from men in the family (e.g. father) - General lack of hope for the future due to immutability of their situation |

**Table B. Key findings for caregivers by country: Risk and protective factors for adolescents engaging with armed groups and reintegrating with communities before, during, and after conflict**

| **Theme** | **Central African Republic** | **Democratic Republic of Congo** |
| --- | --- | --- |
| Pre-conflict | **Risk factors for engaging with armed groups**   - Poor economic conditions of the household - Child having no school or occupation - Social influence of other young people   **Challenges for caregivers**   - Caregiver disapproval of child’s involvement, but did not know until they went missing   - Felt they had little control over their child’s behavior | **Risk factors for engaging with armed groups**  Poor economic conditions of the household   - Indistinct line between child engaging with armed groups out of their own will or being forced to join or kidnapped   **Challenges for caregivers**   - Caregiver disapproval of child’s involvement, fear and sadness |
| Conflict | **Challenges for caregivers**   - Traumatic conflict experiences including children being killed or separated from them   - Lasting effects on their mental health and capacity to care for their families   **Protective factors for reintegration**   - Maintenance of a positive relationship with their child even while they lived with the armed group, providing advice and expressing concern which eventually influenced them to return home, reported mostly by women, some men | **Challenges for caregivers**   - Stigma toward the caregivers from the community and family for child’s engagement with armed group, including cases of imprisonment or other official punishment   - Blame toward mothers by fathers - Increased tension within the household, fighting between partners while child was engaged with armed group, reported by women |
| Post-conflict | **Risks to reintegration**   - Economic stress, unable to adequately provide for children   - Perception that risk of re-recruitment is high due to lack of livelihoods opportunities - Difficulties with child’s behavior (e.g. anger or aggression and disinterest, particularly perceived among boy children)   **Challenges for caregivers**   - Challenges with their own mental health issues and coping as a parent - Difficulties with talking to their child about what happened to them - Successful strategies for talking with their children about their experiences included involving the whole family engaging the child through individual and family conversations   **Needs and areas for support**   - Critical and urgent economic support - Livelihoods opportunities and capital for themselves and their children - Skills and knowledge on child development, child wellbeing, and discipline - Approaches for talking to their children about the conflict | **Risks to reintegration**   - Economic stress, unable to adequately provide for children - Positive change in family dynamics upon child’s reintegration, but variation by gender of caregiver and child (e.g. father not welcoming toward girl child, as reported by women)   **Challenges for caregivers**   - Difficulties with child’s behavior (e.g. anger or aggression and depression, risky or violent behavior outside of the home, perceived among boy and girl children) - Successful strategies for talking with children about their experiences included talking about opportunities for the future, sharing a treat or meal as an entry point to a conversation, talking together while working, giving them a responsibility, and using advice or examples   **Needs and areas for support**   - Critical and urgent economic support - Livelihoods opportunities for their children - Adult literacy and livelihoods training for caregivers - Skills and knowledge on child development and discipline - Approaches for talking to their children about the conflict |
