# Supplementary material for: Drivers of ‘voluntary’ recruitment and challenges for families with adolescents engaged with armed groups: Qualitative insights from Central African Republic and Democratic Republic of the Congo
Source: PLOS Glob Public Health. 2023 May 24;3(5):e0001265. doi: 10.1371/journal.pgph.0001265 (PMC10208483; doi:10.1371/journal.pgph.0001265)
Supplement: Attachment — Submitted filename: Response to Reviewers.docx [file pgph.0001265.s003.docx]

12 December 2022

On behalf of the authorship team, we are pleased to resubmit the manuscript "Drivers of ‘voluntary’ recruitment and challenges for families with adolescents engaged with armed groups: a conceptual framework from Central African Republic and Democratic Republic of Congo" with major revisions. The authorship team thanks the reviewers for their thoughtful and helpful comments which have strengthened the paper. Each of the editorial and reviewer comments are addressed point-by-point in the table below, and revisions have been documented in track changes in the appropriate version.

We look forward to receiving your response to our revision.

Point-by-Point Reviewer Response

| **Comment** | **Response** | **Manuscript revisions** |
| --- | --- | --- |
| *Editorial feedback* | | |
| We've checked your submission and before we can proceed, we need you to address the following issues:  1. We have noticed that you have uploaded Supporting Information files, but you have not included a list of legends. Please add a full list of legends for your Supporting Information files after the references list. | A list of legends for the Supporting Information has been added to the manuscript document after the references list. | See the list of documents in Supporting Information on page 42-43. |
| 2. Thank you for explaining that your full dataset is unavailable due to confidentiality and sensitivity issues. Please note that for studies analyzing data collected as part of qualitative research, authors should make excerpts of the transcripts relevant to the study available in an appropriate data repository, within the paper, or upon request if they cannot be shared publicly. If even sharing excerpts would violate the agreement to which the participants consented, please let us know. | Excerpts from the transcripts relevant to the study are currently included throughout the text presented with the results. As noted in our last submission and correspondence with the editor, the de-identified transcripts can be made available upon request by contacting our Institutional Review Board or the organization’s Monitoring & Evaluation team. It would only violate the agreement within our consent forms and IRB approval if we shared the data in a data repository. We have reached out and confirmed this with our IRB, who agree that the data cannot be made publicly available online through a data repository but could be shared upon request. | Page 44, Availability of data and materials: “The data generated and/or analysed during the current study are not publicly available due to the sensitivity of the data but are available from the corresponding author or the International Rescue Committee Institutional Review Board on reasonable request.” |
| As you will see from the comments, the reviewers and I are impressed with the amount of work that has gone into this manuscript, and the sensitivity shown in presenting the experiences of caregivers and youth. Responding to the reviewers' questions and comments will strengthen the manuscript, and its potential for impact and informing practice. | Thank you for your consideration and for your detailed editorial feedback. | N/A |
| I agree with the reviewers' suggestions concerning clarifying the recruitment, data collection, and analysis methods, and would encourage the authors to complete the COREQ checklist to help with this. I also encourage the authors to include their data collection instruments and final codebook as supplementary materials. | The Methods section has been strengthened based on the feedback from reviewers and in accordance with the COREQ checklist.  Data collection instruments have also been included in Supplemental Information. | Pages 7-16, Methods (Revised manuscript with track changes): Details on recruitment, data collection, and analysis have been added to the Methods section. Please see below for detailed revisions in the manuscript, which are included in the text in track changes. |
| I also agree with the suggestions to revise the results to make the themes and sub-themes clearer to the reader, and that the conceptual figure models are a little hard to understand.  Two of the reviewers provided their comments as an attachment, so please let the editorial staff know if you cannot view the attached comments. | The Results section has been revised to make the themes and sub-themes more clear for the reader. Additional sub-headings have been added to the results and discussion sections to also improve clarity. The conceptual model has also been slightly revised and table with themes by ecological level has been added to the conceptual model to help with interpretation. | Pages 16-31, Results (Revised manuscript with track changes): Headings and subheadings of the results section have been revised to clarify the themes for the reader and results have been slightly reorganized (see track changes in the text).  Page 32, Fig 1: The conceptual model in Fig 1 has been revised for clarity. |
| 1. Please provide a/amend your detailed Financial Disclosure statement. This is published with the article. It must therefore be completed in full sentences and contain the exact wording you wish to be published.  a. State the initials, alongside each funding source, of each author to receive each grant.  b. State what role the funders took in the study. If the funders had no role in your study, please state: “The funders had no role in study design, data collection and analysis, decision to publish, or preparation of the manuscript.”  c. If any authors received a salary from any of your funders, please state which authors and which funders. | The following statement is required in all publications as part of financial disclosure for the grant from the USAID, and therefore must be retained: “This research was made possible through a grant by the United Stated Agency for International Development (720FDA19GR00218). The contents are the responsibility of the International Rescue Committee and do not necessarily reflect the views of USAID or the United States Government.”  The grant was received by the International Rescue Committee and was not received by individual authors. However, several authors received a salary from this grant, which has been added to the statement. | Page 43, Funding (Revised manuscript with track changes): “This research was made possible through a grant by the United Stated Agency for International Development (720FDA19GR00218). The contents are the responsibility of the International Rescue Committee and do not necessarily reflect the views of USAID or the United States Government. AB, YA, JUW, AN, PG, BK, and KF received partial funding for their salaries from the USAID as part of this grant. DO was funded by the IRC-Zolberg Fellowship.” |
| 2. Since your data is not available for proprietary reasons, please explain via email why the data is not available. Please also include the contact information for the third party organization that should be contacted should other researchers want to request access to this data and please include the full citation of where the data can be found. We also request that you verify with us via email that any researcher will be able to obtain the data set in the same manner that the you have obtained it. If you feel you are unwilling or unable to adhere to this policy, please explain your reasons by return email and your exemption request will be escalated to the editor for approval. Your exemption request will be handled independently and will not hold up the peer review process, but will need to be resolved should your manuscript be accepted for publication. One of the Editorial team will be in touch if they require more information. | As stated in the statement on Availability of data and materials, the data generated and/or analyzed during the current study are not publicly available due to the sensitivity of the data but are available from the corresponding author on reasonable request. The data cannot be published publicly through a third party organization because they contain detailed information regarding the involvement of minors in armed forces and armed groups, including reporting on specific individuals and events which could result in a breach of confidentiality and risk of harm to respondents and their communities. This decision is supported by Reviewer 1 in their second comment in the table below. However, we verify that any researcher will be able to obtain the data set in the same manner that it was obtained by contacting the International Rescue Committee. We will send a verification with further justification by email. | N/A |
| *Reviewer 1* | | |
| First, thank you for your dedication to this population and the clear and obvious commitment displayed for their safety throughout the research process. | We thank you for your thoughtful comments and suggestions, which have helped us to strengthen the manuscript. | N/A |
| I agree with the authors' decision to not make the primary data publicly available based on concerns for safety and ethics. That said, in light of the journal's orientation towards replicability and transparency, it might be worth considering whether the qualitative instrumentation (interview guides) or analytical tools (code books, coding trees, etc) should be provided. | Thank you for your confirmation of our decision not to share the primary data publicly. We agree with your suggestion to share the qualitative instruments and have included them as Supplemental Information with the submission. | N/A |
| I would suggest pulling the contextual study setting information out before the methods section. Currently, this section feels to unnaturally break up the methodological information with historical and contextual information better suited for earlier in the manuscript. | This sub-section has been pulled out and inserted into the Introduction section in accordance with this comment. | See Page 5, Introduction, Line 114 (Revised manuscript with track changes): The sub-section “Study Setting” has been moved to the Introduction. |
| I would also suggest providing all relevant information related to participant identification and recruitment in one place. Currently, there is rather vague recruitment information presented in "Study Participants". Additional information about recruitment and site selection were included in "Data Collection" and "Ethical and Safety Considerations". | The “Study participants” section has been revised to include all relevant information on participant identification, recruitment, and sampling. | See Page 10, Study participants, Line 224 (Revised manuscript with track changes): “To better understand the experiences of adolescents and their families living in conflict settings, in-depth interviews were conducted with adolescents formerly engaged in armed groups or at risk of joining and their caregivers. Inclusion criteria for the study were …” (See full revision in the text.) |
| Recruitment challenges were noted and changes to the process were mentioned. However, it is unclear what these changes entailed or how they improved recruitment. | More detail on these changes and how they improved recruitment has been added to the text and moved to the section on Study participants for consistency. | See page 11, Study participants, Line 264 (Revised manuscript with track changes): “Purposive sampling was used to recruit study participants within each site. Initial sample sizes were decided based on the caseload of the [REDACTED] Child Protection teams in each site, with prioritization of adolescents formerly engaged with armed groups and older adolescents, given the aims of the study. However, after some initial challenges…” (See full revision in the text.) |
| I see that data were coded by one member of study team. Does this indicate that all interviews were coded by the same research team member or that each transcript was coded by only one person? | Each transcript was coded by only one person, with exception of 5 transcripts (5%) which were randomly selected and double-coded at the start of the coding phase. The research team then met to discuss any discrepancies and differences in the coding application and grounded codes, and the coding criteria was revised as needed. This has been further explained in the text to provide clarity. | Page 15, Data analysis, Line 369 (Revised manuscript with track changes): “Data were coded by two members of the research team, with double-coding conducted on a random 5% of the articles. After an initial round of coding, the study team met to review the grounded codes and discuss and resolve any discrepancies and coding criteria was adjusted.” |
| As presented, Tables 2 and 3 are difficult to follow. I am unsure if differences in word choice are reflect true nuance in the data, or if these are simply different phrases used to describe similar phenomena. For instance, is "no access to basic needs or livelihoods" distinct from "no access to income" and "caregivers unable to provide for needs". | The text in the tables has been revised to provide consistency where they are describing similar phenomena, and clearer discrepancy where they are meant to reflect nuance in the data. | See Supplemental Information, Tables 2 and 3: For example, in the DRC column under “Readjusting to life at home”, “No access to income” has been revised to read “No access to basic needs or livelihoods (e.g. previous source of income was through association with armed group)” to be consistent in wording with a similar finding from CAR but elaborate on a point that is nuanced for the DRC. |
| Table 2 seems to suggest that in CAR a belief or affiliation with the group drives engagement, while the opposite seems to be true in the DRC (that a lack of that affiliation drives engagement). However, the reading of the results section seems to present a different story. Namely, that this affiliation just isn't a salient factor for engagement in the DRC. | It is correct that this affiliation isn’t a salient factor for engagement in the DRC, as described in the results. Table 2 has therefore been revised to remove “No affiliation with cause of the armed group and general dislike of armed groups” from the column for DRC to avoid confusion. | See Supplemental Information, Table 2: In the DRC column under “Drivers of engagement with armed groups”, “No affiliation with cause of the armed group and general dislike of armed groups” has been removed |
| I might suggest thinking through whether tables of the nature of Tables 2 and 3 are really the most appropriate way to present this qualitative data. | We received comments in a previous round of review that a summary table was needed at the start of the results section to provide clarity on key findings because of the length of the results. However, given the multiple comments about the tables from reviewers in this round, we have decided to put the tables into Supplemental Information where readers could view them if desired. | Tables 2 and 3 have been moved to Supplemental Information. |
| My biggest concern with this manuscript relates to the analytical process. Currently, the methodology reads as if themes were developed. But themes do not seem to be clearly presented. What appears under the heading of "theme" in Tables 2 and 3 appear to align better with the concept of theme categories related to the temporality lens the authors apply in the presentation of results. In the results section, themes are not clearly delineated, but the data seems to simply be described. Likewise, pieces of the analytical process seem to be borrowing from grounded theory. But without a theoretical framework for the analysis clearly stated, it is difficult to determine whether the analytical process described is appropriate. | The analysis used a chrono-ecological model to examine the different levels at which factors influence recruitment and reintegration over time, to capture the different pre-conflict, conflict, post-conflict (time) experiences and drivers at different socioecological levels (Institutional, Societal, Community, Family / Interpersonal, and Individual). Using this a priori framework, themes were developed within each time point and ecological level using grounded theory. The data was collected and analyzed using a narrative inquiry approach, which aims to capture respondents’ lived experiences and center the meaning that individuals give to a shared story (Esposito & Evans-Winters, 2022).  More information on the framework used for the analytical process has been added to the Methods section under “Data analysis”, and the themes and headings in Tables 1 and 2 and in the Results and Discussion sections have been revised to clarify this framework and to add nuance for context-specific findings. | Page 15, Data analysis, line 364 (Revised manuscript with track changes): “The research applied a narrative inquiry approach in data collection and analysis, which aims to capture respondents’ lived experiences and present a shared story which depicts themes and patterns based on the meaning and understanding that individuals give them (32). Therefore, data are presented descriptively to depict the patterns in these shared stories in terms of their narrative structure and content.  Data were coded using combination of a priori and inductive coding. An initial coding structure was developed using an adapted chrono-ecological framework (33,34), which examined the factors that influenced adolescent’s recruitment and reintegration at different levels of the ecological model (institutional, societal, community, interpersonal, and individual) over time (pre-conflict, conflict, and post-conflict). Using grounded theory, new codes were then added based on emergent themes and patterns in participants’ responses for each of these levels and time points within the chrono-ecological model. Detailed coding trees were then drafted with the combined deductive and inductive codes for each concept explored in the data and for each type of respondent, and a codebook was developed based on the coding tree.”  Pages 16-31, Results (Revised manuscript with track changes): Headings and subheadings of the results section have been revised to clarify the themes for the reader and results have been reorganized (see track changes in the text).  Page 32, Fig 1 (Revised manuscript with track changes): The conceptual model in Fig 1 has been revised for clarity. |
| Overall, the results seem to be getting at an important and potentially useful conceptual framework. However, the stories of the different groups get lost in the sheer volume of data being presented and the lack of organization to guide that presentation. There are both gendered and country-specific lenses being applied to the data. But the results often jump from those directly engaged to those at-risk of being engaged to caregivers and often back to adolescents of some kind. It's challenging to follow any one thread or narrative in its current form. I would suggest a more structured presentation utilizing clear headers and subheaders. For instance, you could discuss cross-cutting ideas (spanning both CAR and DRC), then move to discussing ideas that were contextually specific or distinct. Given that the paper's Figure 1 depends on data from all stakeholder groups, it likely does not make sense to break this paper out into more focused publications. However, I do recommend considering an organization approach that makes this volume of data more reader-friendly. | The results have been reorganized and headers and sub-headers revised to provide clarity to the organization of the paper.  Findings were similar for many themes in both DRC and CAR, which is what prompted us to combine them into one paper. For the themes where findings were distinct by context, the differences in each country are highlighted in the text. Given the similarities between the two countries and that the journal formatting guidelines permit only 3 levels of headings, we decided not to break the findings down into headings or sections based on country. However, where differences by group were important, these are highlighted in the sub-headings and in the conceptual model in Figure 1 to pull them out.  In addition, the tables (now moved to the Supplemental Information section) provide summaries by country and time point so as to allow for direct comparison of the two contexts. | See above revisions. |
| *Reviewer 2* | | |
| All my comments are included in the attachment. No other comments are written here. |  |  |
| Summary: This manuscript seeks to gain greater understanding on the drivers of voluntary recruitment from the perspectives of adolescents and their caregivers, as well as to explore barriers and facilitators for reintegration of CAAFAG. Using qualitative methods and interviews in 2 country settings, DRC and CAR, the findings will contribute to better understanding of the needs and experiences of children and their families and help inform more comprehensive programming models. This paper adds important perspective from adolescents and caregivers to understand the push and pull factors for recruitment and reintegration of CAAFAG. The manuscript would benefit from revision, to provide more clarity. These are detailed in the comments below | We thank you for your thoughtful comments and suggestions, which have helped us to strengthen the manuscript. | N/A |
| **Methods.**  1. The authors mention the formation of a external technical advisory group. Additional information on the participants and involvement of the TAG is needed. For example, are members of the TAG from CAR and DRC? Are families, former CAAFAG and/or local organizations included in the TAG? How often did the TAG meet and what was their focus? | The TAG consisted of 8 global, regional, and local experts, including members of local organizations and local service providers. Procedures and tools were also reviewed by the local community networks that were used to identify and recruit participants for the research. Further detail has been added to the text explaining the technical advisory group. | Page 7, Methods, line 167 (Revised manuscript with track changes): “All research methods and tools were also reviewed by an external technical advisory group (TAG) convened for the project, which was made up of eight global, regional, and local experts on research and programs for children associated with armed forces and armed groups (CAAFAG), including representatives of academic institutions, UN organizations, and international and national governmental organizations and service providers. The TAG met periodically throughout the research design and implementation and provided feedback on research tools and methodological and ethical procedures, which were adapted based on their suggestions. In addition to the TAG, the protocol and tools were reviewed by local service providers who provided inputs and advised on contextually relevant risk mitigation procedures, measures for ensuring confidentiality and privacy, informed consent, and referral procedures.” |
| 2. The study included interviews with caregivers of adolescents. Are these caregivers the same caregivers of adolescents who participated in the study? | Given that community-based child protection committees and local service providers recruited study participants by identifying families who they work with in the community, many caregivers who participated in the study were the same caregivers of adolescents who participated in the study. However, this was not part of eligibility criteria for caregivers to participate. This clarification has been added to the text. | Page 10, Study participants, Line 247 (Revised manuscript with track changes): “Caregivers did not have to be the caregiver of a child participating in the research to also be included in the study.” |
| 3. In DRC, were participants of Rwandan and Congolese ethnicity? | We did not collect data on ethnicity. | N/A |
| 4. On page 9, lines 199-203, the authors state that there was difficulty identifying adolescent girl respondents and at-risk respondents in CAR. Though they state that they increased sample size in CAR and altered the sample in DRC, it is not clear what changes were made to address the challenge. How does increasing the sample size for a hard to access population resolve the challenge of identifying participants? What changes were made to recruitment in DRC? | After initial challenges in CAR, we adapted our sampling approach and added additional interviews to target more adolescent girls and to increase the sample size overall. More detail on these changes and how they improved recruitment has been added to the text and moved to the section on Study participants for consistency. | See page 11, Study participants, Line 264 (Revised manuscript with track changes): “Purposive sampling was used to recruit study participants within each site. Initial sample sizes were decided based on the caseload of the [REDACTED] Child Protection teams in each site, with prioritization of adolescents formerly engaged with armed groups and older adolescents, given the aims of the study. However, after some initial challenges…” (See full revision in the text.) |
| 5. The authors provide a clear description of the lifeline methodology. Some information on the types of questions or thematic areas that were used to guide the IDI and application of the lifeline approach will help the reader understand the type of information that the researchers sought to understand from the different types of participants (location, gender, type of adolescent, caregiver). What type of interview was done with the caregivers – i.e., also a lifeline approach? | More information on the questions and types of information collected for both adolescents and caregivers was added to the text for clarification. | Page 13, Data collection, line 302 (Revised manuscript with track changes): “The guide included questions on their household dynamics and experiences during each of these periods and how they changed over time, as well as why they did or did not engage with armed groups and how they reintegrated into their families and communities. For caregivers, traditional semi-structured interview guides were used to understand caregivers’ perceptions of their children’s experiences during these periods, as well as their own experiences and challenges at the same time. Demographic information including age, gender, disability level, displacement status, marital status, and household composition were also collected from all respondents.” |
| 6. How did the staff ensure safety of the participants in the study, given that recruitment focused on CAAFAG? Were there any concerns of increasing stigma against children or their caregivers? | Significant care was given to ensuring the safety of research participants, given the sensitive nature of the study. Ethical and safety procedures are described the Methods under “Ethical and safety considerations” (Page 13).  In addition, detail was provided on the participant recruitment process and how the research team worked with local community networks for child protection and identification of CAAFAG, which helped to ensure that procedures used in the study were safe and appropriate for the context. (See page 10, the paragraph starting on line 235.) | See the additional detail added to the Methods, Study participants, Page 11 line 249: “Sites for the study were selected based on the presence of [REDACTED] Child Protection case management services…” (See full revision in the text.) |
| 7. How were data managed during the data collection to ensure safety, such as the recordings of interviews, use of ID codes, and transfer of data? | Additional information on data management and protection has been added to Ethical and Safety Considerations in the Methods section (page 13). | Page 15, Ethical and safety considerations, Line 352 (Revised manuscript with track changes): “Participants were assigned an ID number when enrolled in the study, which was included on the informed consent forms and used to link audio recordings, transcripts, and any other documents from the interviews. Data files including audio files, transcripts, and scanned documents were saved in password-protected files on locked computers held by the research managers in each site. Once audio recordings were saved to the computer, they were deleted from the audio recording device. Participant ID, gender, age, and village were included on transcripts, but all personally identifying information, including the names of specific towns and armed groups, were removed from transcripts, notes, and drawings during transcription. In consultation with local partners, presentation of analyses removed any other information that could lead to a breach in confidentiality, such as the name of the research site, including quotes within this paper.” |
| 8. Did any of the analysis examine the gender differences between CAAFAG? The results tables mention this difference where relevant. If the analysis focused on this when examining differences in coding, please make mention of it in the methods. | Yes, analyses focused on variation by demographic characteristics including gender and age, as well as other characteristics. Where differences were identified, they have been presented in the text and results tables.  This approach was added to the Methods for clarity. | Page 16, Data analysis, Line 375 (Revised manuscript with track changes): “Analyses focused on variation by demographic characteristics, examining differences in emergent themes by gender, age, and other characteristics.” |
| Results.  1. Page 14, Line 283-284, Did the adolescent join the same armed group that killed his two older brothers? | In this case the adolescent boy’s brothers were killed by the opposing armed group, so he joined the other armed group to avenge his brothers.  This has been clarified in the quote. | Page 19, line 471 (Revised manuscript with track changes): “Yes, the [opposing armed group] killed my two older brothers in [village]. … Which made me angry and decide to join the armed group to avenge my two older brothers.” |
| 2. Editing: Line 435, change DRF to DRC | Thank you for catching this typing error, it has been addressed. | Page 24, line 586 (Revised manuscript with track changes): “Several boys and girls in both CAR and DRC…” |
| Discussion:  1. Figures: The conceptual frameworks are not clear depictions of the analysis and require more explanation (and possible revision) for the reader to understand what the authors are depicting and how the reader is meant to interpret and apply them. | The conceptual framework has been revised to clarify the chrono-ecological model. The model has been further explained in the text for clearer interpretation. | Page 32, Discussion, paragraph 1 and Fig. 1 (Revised manuscript with track changes): See revised figure and text. |
| 2. Lines 691 – 694: Do the cited papers reference parent’s mental health as a factor associated with children’s recruitment or abduction into armed groups, or that they are associated with harmful practices more broadly? I am not convinced that the data in the paper support parent’s mental health as a reason for children to join or consider joining armed groups. The quotes shared reference negative home environments without explaining what about the environment is negative. If there is additional information describing the home environment to add from the transcripts, it would be beneficial. If not, the authors need to make the rationale for their argument clear, and grounded in current evidence. | The data in the paper show that adolescents who perceived a negative family environment, including negative family dynamics and experiences of physical and emotional maltreatment and neglect, mentioned this as a driving factor for their engagement with the armed groups. This is apparent in quotes such as the following from page 23 (Revised manuscript with track changes): “One 17-year-old girl in the DRC shared about her relationship with her parents before she engaged with the group: “It was negative because, when one goes wandering, it is not because there is nothing to do a home. There are chores at home, but you go. If they are pouring out abuse on you, you decide to go wandering.”  The discussion is making the link between the family environment, the parent-child relationships, child mental health, and child recruitment. The papers cited show that child maltreatment and neglect influence the mental health and behavior of a child. Similarly, caregivers’ own mental health impacts the mental health and caregiver of a child, and the data show that caregivers continue to struggle with their mental health related to conflict experiences and daily stress. Therefore, while parent’s mental health is not a reason for children to join, it is linked to the parent-child relationship and the child’s mental health and behavior, which are all related to their engagement with armed groups.  The results have been restructured in a way that better supports this rationale, and the text in the discussion has elaborated on this point to provide more clarity. | Page 23, Results, Interpersonal level: Household dynamics as a push or pull factor, line 564 (Revised manuscript with track changes): “Several adolescent respondents reported that the negative situation with their family in their home—for example, adolescents described both verbal and physical abuse, as well as a general unsupportive family dynamic and feeling unhappy or unwelcome—drove them to affiliate with the armed group. This was reported mostly by adolescent girls in the DRC.”  Page 34, Discussion, Interpersonal Factors, line 875 (Revised manuscript with track changes): “Girls who previously engaged with armed groups also more often reported having particularly negative and harmful relationships with their caregivers, including emotional and physical maltreatment and neglect, which drove them to leave home to engage with the armed group.”  Page 35, Discussion, Interpersonal Factors, line 893 (Revised manuscript with track changes): “While there is little existing evidence on the specific experiences of parents or recruited children, recent studies have demonstrated that caregivers’ own mental health issues are associated with neglect and harmful practices toward children (43–46), in turn negatively affecting child mental health and wellbeing (47,48). This perceived lack of parental involvement in a child’s life and child maladjustment can be risk factors for engagement with armed groups (4).” |
| 3. What are the limitations of this study, beyond it not being generalizable? | More information on limitations has been added to the Conclusion section. | Page 37, Conclusion, line 923 (Revised manuscript with track changes): “The findings from this study are based on qualitative interviews from a small sample of adolescents and caregivers in only two conflict settings in central Africa and therefore not generalizable to all active conflict settings. While the sampling approach involved the recruitment of study participants through established community networks…” |
| 4. The authors do not comment on the importance of the community/social environment to push and pull factors for recruitment and reintegration. Based on the interviews, did the authors learn anything about this? If not, do the authors recommend additional information or focus on the level of the ecosystem: the social/community-adolescent and social/community – caregiver relationship? | Community and social push and pull factors are indeed included in each time period within the Results where these factors came up in the interviews, as well as in the reflections in the Discussion. The re-organization of Results and addition of sub-headings to both the Results and Discussion sections should help to clarify where these factors are highlighted. | See the relevant sections in the text, including (Revised manuscript with track changes):   - Results, Pre-Conflict, Community level: The influence of social networks, page 20 - Results, Post-Conflict, Community level: Gendered experiences of reintegration and stigma for girls and boys, page 28 - Discussion, Community level factors, page 33 |
| *Reviewer 3* | | |
| The authors explore well debated phenomenon of child and adolescent involved in armed conflicts, particularly the increasing self-enrolment or “voluntarily enrolment” which is contested by several scholars who rethink the concept of child soldiers from the perspectives of African rites of passage (initiation) to adulthood, which starts from twelve to full adulthood (Honwana 2006; Van Rooyen et al. 2006). The authors further explore the prevention of child and adolescents’ recruitment, their use as soldiers, re-remobilisation, their reintegration from the perspectives of their caregivers, and the extent to which family can lessen their chance of re-recruitment. The findings reveal a nexus of factors that maintain the vulnerability of boys and girls to recruitment and re-recruitment. Those factors include:  • Traumatic experiences and economic hardships keep children and adolescents vulnerable.  • Erosion of family protective family relationships endangers meaningful reintegration.  • Familial support would prevent re-recruitment.  • A better understanding of children’s experiences in armed conflict can incentivise resiliency of child soldiers and their support by caregivers.  • more comprehensive programming models can be developed to prevent voluntary , etc.  Lastly,  • the authors highlight the necessity of a family-based approach to ameliorate adolescent resilience to recruitment by armed groups.  • They advocate additional programs of caregivers’ support in armed conflict settings to increase their own mental health and family functionality.  • Better support structures are also called for to prevent recruitment, facilitate a successful reintegration of returnee young soldiers, and minimise the danger of re-mobilisation, tec.  While the manuscript shows a lot of potential, especially the finding if brings forth, the paper has serious weaknesses which include:  • Lack of clear research design to outline how the research will be conduct and who will be participating and what they will be contributing to generate knowledge.  • The conceptual framework is not well defined and explained as far as voluntary recruitment of different age groups are concerned.  • There is little engagement with existing legal frameworks and literature pertaining to child and adolescent recruitment.  • The lack of a well explained theoretical framework makes it hard to follow the orientation of the inquiry.  Additional comments and advice are subsequently provided to ameliorate the scientific value of this paper.  The paper requires major corrections. | We thank you for your thoughtful comments and suggestions, which have helped us to strengthen the manuscript. We have tried to address them where possible, and have provided explanation for those comments that we feel cannot be addressed within the scope of this paper. |  |
| **Title:**   - The use of “conceptual framework” appears problematic in the title, because there is no discussion of the concept of voluntary recruitment before the findings. - Voluntary recruitment and the use of the concept of child soldiers is hotly disputed in the context of African cultural rites of initiation which assumes the transition from childhood to adulthood for youngsters aged between twelve and 24 or even above (for uncircumcised adults) in the Zulu, Xhosa, Ndebele culture, etc. - There is no discussion on the legal and humanitarian aspects of voluntary recruitment. - As a result, the authors use child recruitment and adolescent recruitment without prior delineation of the parameters of these concepts.   Such a compounded approach of the problem creates some difficulties which I will explain, and that could only resolved by separating the cases of child recruitment and adult recruitment according to the legal domestic and international frameworks in this matter. | The conceptual framework in the title of this paper references the theoretical framework (Fig. 1) that was developed based on a chrono-ecological analytical model and grounded theory.  The concept of voluntary recruitment is presented in the introduction and includes its conceptualization for this paper, as well as a brief overview of child developmental theories on voluntary and forced recruitment. A sub-heading has been added to the Introduction to better clarify where this concept is first discussed, and a sentence has been added with more context from international humanitarian law. The national legal frameworks regarding child recruitment are further presented in the sub-section in the introduction titled “Study setting”, which has been moved from the Methods to the Introduction.  This paper does not focus on adult recruitment and for this reason the concept is not further defined. | For a presentation of the concept of ‘voluntary’ recruitment, see the definition on Page 3, Introduction, ‘Voluntary’ recruitment of children to armed groups, line 59 (Revised manuscript with track changes).  A line has been added on international human rights and humanitarian law on Page 4, line 75 (Revised manuscript with track changes): “While in many contexts including the DRC and CAR adolescents are considered adults within society based on cultural rites of passage and initiation, international human rights and humanitarian law prohibits any recruitment or use of persons under the age of 18 by armed groups and bans the forced recruitment and participation of children under 18 in state militaries, and no distinction is made between ‘voluntary’ and forced recruitment of this age group (6).”  For national legal frameworks, see Page 5, Introduction, Study setting, beginning line 114 (Revised manuscript with track changes).  For further description of the framework, see Page 32, Discussion, line 810 (Revised manuscript with track changes): “The framework summarizes the influence of the institutional, social, community, and interpersonal factors that influenced the individual child’s engagement with armed groups and reintegration into the community before, during, and after the conflict. This framework shows the drivers of recruitment from the perspectives of both the adolescent respondents and their caregivers at different levels of the ecological framework, with the size of each level denoting its relative importance as determined by occurrence in the transcripts. This separate depiction of adolescents and caregivers is not meant to contrast their perspectives but rather to draw out the central factors that both sets of respondents perceive to influence children to engage with armed groups, and to understand the overlapping needs of families living in conflict settings that affect household dynamics and erode the protective family environment.” |
| **Abstract**  *“The number of children voluntarily engaging with armed groups is also rising, despite increasing efforts to prevent this grave human rights violation”.*  *“In-depth interviews were conducted with 74 adolescents (44 boys and 30 girls) and 39 caregivers (18 men and 21 women) in two distinct conflict settings”*  **Comments:**  It is not clear whether the concerns applied to both children and adolescents. The authors should be more specific whether “voluntary recruitment and challenges” and provide the age range of the 74 adolescents as done for the caregivers and explain to which extent the problem *being addressed is falls under humanitarian or legal* | The fact provided in the first sentence regards both children and adolescents broadly. The sentence has been revised to clarify.  The research involved adolescents, and the age ranges have been added to the abstract. | Page 2, Abstract, line 23 (Revised manuscript with track changes): “The number of children and adolescents voluntarily engaging with armed groups…”  Page 2, Abstract, line 30 (Revised manuscript with track changes): “In-depth interviews were conducted with 74 adolescents (44 boys and 30 girls) ages 14 to 20 years and 39 caregivers (18 men and 21 women) ages 32 to 66 years in two distinct conflict settings: the North Kivu, Democratic Republic of Congo and Ouham-Pendé, Central African Republic.” |
| *“The findings examine the unique perspectives of adolescents engaged with armed groups and their caregivers to understand how conflict experiences, economic insecurity, and social insecurity influence children’s engagement with armed groups and reintegration with their families”.*  **Comments:**  The author should be careful in the use of children and adolescents which should not be used interchangeably, because the dynamics of their recruitment and the challenges they are confronted with are not weighed in the same way as far as the lawfulness of enrolment and humanitarian implications of recruitments are concerned. **AGE MATTERS A LOT IN AN INQUIRY OF THIS NATURE.** | Thank you for noting the importance of this distinction. The use of the term adolescents has been clarified and revised throughout the paper. | The term “adolescent” has been used in place of children throughout the paper, where the distinction is relevant. |
| *By better understanding the experiences of children enduring recruitment and how to support caregivers of those children, more comprehensive programming models can be developed to adequately prevent voluntary recruitment and promote successful reintegration, enabling children to reach their full potential.*  **Comments:**   - What will be the legal and humanitarian ground for the prohibition of adolescents to serve in armed groups and in the regular armed forces? - It is not helpful to leave the readers guessing about the why the authors make an issue with the participants of adolescents aged above 18 to join fighting forces. - For instance, in line 150, the authors observe: “Government-led armed forces stopped enlisting 150 children in 2003, and the DRC government…”. - What do “Government-led armed forces” entail? - There is no legal argument about the prohibition age for participations of adolescents in armed groups. | National and international legal frameworks prohibit the recruitment and use of individuals under the age of 18 in any armed force or armed group, including government forces. This is described in the Introduction section on ‘Voluntary’ recruitment of children to armed groups (page 3).  This study includes adolescents over the age of 18 because they can provide information on their experiences and perceptions of the recruitment that they experienced before the age of 18.  Revisions have been made to the text to clarify the above points. The use of the term adolescents has been clarified and revised throughout the paper. | Page 4, line 75 (Revised manuscript with track changes): “While in many contexts including the DRC and CAR adolescents are considered adults within society based on cultural rites of passage and initiation, international human rights and humanitarian law prohibits any recruitment or use of persons under the age of 18 by armed groups and bans the forced recruitment and participation of children under 18 in state militaries, and no distinction is made between ‘voluntary’ and forced recruitment of this age group (6).”  For national legal frameworks, see Page 5, Introduction, Study setting, beginning line 114 (Revised manuscript with track changes).  Page 10, Study participants, line 239 (Revised manuscript with track changes): “Older adolescents ages 18-20 were included so as to gather information about their experiences when recruited as adolescents under the age of 18.” |
| *85 deterrent and protective factor for children. One recent study underscored the perceived lack of parental*  *86 involvement in the child’s life as a critical factor for engagement with violent extremism in Africa (4).*  *87 Other studies have explored how family cohesion and positive parent-child relationships could be a*  *88 source of resilience in crisis settings (12–14) and therefore a possible deterrent for recruitment, while an*  *89 emotionally supportive family environment can curtail retention in armed groups and positively influence*  *90 reintegration (15,16). Nevertheless, a deep understanding of the experiences of families living in conflict*   91 *settings with armed recruitment is lacking.*   The authors have limited understanding of the multifaced push and pull factors that sustain re-mobilization in fighting forces besides causes of child recruitment, namely participating in the community self-defense initiatives in the East DRC to protect their communities against foreign invaders. | Other push and pull factors are presented in the Introduction and Results, including push and pull factors for child recruitment and re-recruitment at the institutional, societal, community, and interpersonal levels. This study addresses a critical gap in evidence on household dynamics and parent-child relationships for families experiencing recruitment, and for this reason it focuses primarily on the interpersonal level and how factors at the household level influence and are influenced by the conflict and child recruitment. This approach is outlined in the Introduction, Methods, and Results sections, and is reiterated in the Discussion.  Factors such as community and family self-defense are addressed in the Results section relative to both DRC and CAR. | N/A |
| *103 Qualitative data were collected across 8 research sites in conflict-affected communities in CAR*  *104 and DRC where child recruitment is common.*  *What are the five research sites?* | The specific names of the research sites are not provided to protect the confidentiality and safety of the communities that participated in the study. As mentioned in the introduction under Study setting (page 5, Revised manuscript with track changes), research activities were conducted in Ouham-Pendé, CAR and North Kivu, DRC. | N/A |
| **Introduction**  **Comments:**   - The author should provide the outline of the paper before discussing the methods.   The methodological approach should also be included in the introductory part to guide the readers towards what they will find in this study. | The methodological approach and objectives of the paper are provided in the Introduction on page 5, line 105 (Revised manuscript with track changes). | N/A |
| **Methods**  *103 Qualitative data were collected across 8 research sites in conflict-affected communities in CAR*  *104 and DRC where child recruitment is common.*  **Comments:**   - The methodological approach used by the author is flawed. - The author should explain which design was used to conduct this inquiry. - They should also explain the methods used. - The authors suggest that their will use “visual narrative”, unfortunately it only mentioned in the abstract.   Was “visual narrative” part of the methodological strategies? | The first sentence of the Methods section has been revised to include more detail on the methodological approach used.  Significant detail on the visual narrative technique, including other studies where this methodology has been used, is provided in the existing section on Data collection within the Methods. See Page 12, Methods, Data Collection (starting on line 291, Revised manuscript with track changes). | Page 7, Methods, line 163 (Revised manuscript with track changes): “In-depth qualitative interviews were conducted with adolescents and caregivers across 8 research sites in conflict-affected communities in Ouham-Pendé, CAR and North Kivu, DRC where child recruitment is common.” |
| *138 Research activities were conducted in five research sites in North Kivu, DRC*  What are the names of the 8 research sites, they could be useful for the readers and fellow researchers? | Please see the response above regarding the removal of the specific names of the research sites to protect the confidentiality and safety of the participating communities. | N/A |
| **156 Study participants**  *157 To better understand the experiences of adolescents and their families, in-depth interviews were*  *158 conducted with 74 adolescents (39 in DRC and 35 CAR) from ages 14 to 20 years, with respondents*  *159 being 18 years of age on average in CAR and 17 years of age on average in DRC*  **Comments:**   - Recruitment with the two different age groups (p. 157 and 159) are not regulated in the same way by the international legal frameworks pertaining to child soldiers. - How were participants recruited?   What sampling techniques were utilised in that process? | As mentioned in the response above, this study includes adolescents over the age of 18 because they can provide information on their experiences and perceptions of the recruitment that they experienced before the age of 18.  Detailed information on sampling and recruitment of study participants has been added to the Methods sub-section on Study participants (starting on page 11, line 249). | Page 10, Study participants, line 239 (Revised manuscript with track changes): “Older adolescents ages 18-20 were included so as to gather information about their experiences when recruited as adolescents under the age of 18.”  See the additional detail added to the Methods, Study participants, Page 11 line 249 (Revised manuscript with track changes): “Sites for the study were selected based on the presence of [REDACTED] Child Protection case management services…” (See full revision in the text.) |
| The statement below refers to the Paris Principles.  *Association with armed groups could include any engagement including but not limited to*  *164 living with the armed groups, engaging in combat, and conducting other tasks such as cooking cleaning,*  *165 as well as living at home and traveling to the group’s base to trade with or sell goods to them*  2.1“**A child associated with an armed force or armed group”** refers to any person below 18  years of age who is or who has been recruited or used by an armed force or armed group in any capacity, including but not limited to children, boys and girls, used as fighters, cooks, porters, messengers, spies or for sexual purposes. It does not only refer to a child who is taking or has taken a direct part in hostilities.  **Comments:**   - Are the “children” and “youths” here identified as CAAFAG?   This definition does not apply to persons who have crossed the threshold age of prohibition to be recruited as soldiers. | We agree that this definition of engagement with armed groups is in accordance with international law. This has been clarified in the text.  As described in the Methods subsection on Study participants (page 10), participants included both CAAFAG and non-CAAFAG.  The issue regarding the age of adolescent participants has been addressed above. | Page 10, Methods, Study participants, line 233 (Revised manuscript with track changes): “In accordance with definitions set out in international law, association with armed groups could include any engagement including but not limited to living with the armed groups, engaging in combat, and conducting other tasks such as cooking cleaning, as well as living at home and traveling to the group’s base to trade with or sell goods to them.” |
| **Data collection**  *To facilitate participatory*  *184 conversations with the adolescents, the research team employed a narrative life-line technique.*  **Comments:**   - Who made the research team? - What was their role and significance in this inquiry? - What was their competence to engage with this category of research participants? - Was the research team made of the actual authors of the manuscript?   The research methodological approach should also deal with these questions. | Details on the research team including data collection staff, qualifications, and how they were trained can be found under the Methods subsection on Ethical and safety considerations, starting on line 342: “Gender-matched data collection staff were hired locally and were screened for prior experience in child protection and/or psychosocial counseling for children….” | N/A |
| *196 space of the respondent’s choosing. Audio recordings were then transcribed and translated into French*  *197 and English by transcribers and reviewed and verified by the research assistants who conducted the*  *interview, as well as the data collection supervisors. The completed*  *198 life line diagrams were photocopied*  *199 and translated into French and English.*  **Comments:**   - Who were research assistants? - Were they part of the authors? - What were their competence? - The authors should include information about the research team and research assistants earlier under the methodological approach.   The research methodological approach should also deal with these questions. | As mentioned above, details on the research team including data collection staff, qualifications, and how they were trained can be found under the Methods subsection on Ethical and safety considerations, starting on line 342: “Gender-matched data collection staff were hired locally and were screened for prior experience in child protection and/or psychosocial counseling for children….”  Data collection supervisors from both sites were included as authors on this manuscript. | N/A |
| **Data analysis:**  *233 Transcripts and photographs of the life-lines were coded and analyzed using the qualitative*  *234 analysis software Dedoose. Data were analyzed using combination of a priori and inductive coding.*  **Comments:**   - What happened after coding? - Should thematic analysis be applied after coding? - Content analysis and discourse analytical methods could have been utilized to strengthen the interpretation of empirical data used in this inquiry. - How was the reliability of coding and data analysis addressed? | The analysis used a chrono-ecological model to examine the different levels at which factors influence recruitment and reintegration over time, to capture the different pre-conflict, conflict, post-conflict (time) experiences and drivers at different socioecological levels (Institutional, Societal, Community, Family / Interpersonal, and Individual). Using this a priori framework, themes were developed within each time point and ecological level using grounded theory. The data was collected and analyzed using a narrative inquiry approach, which aims to capture respondents’ lived experiences and center the meaning that individuals give to a shared story (Esposito & Evans-Winters, 2022).  More information on the framework used for the analytical process has been added to the Methods section under “Data analysis”.  Each transcript was coded by only one person, with exception of 5 transcripts (5%) which were randomly selected and double-coded at the start of the coding phase. The research team then met to discuss any discrepancies and differences in the coding application and grounded codes, and the coding criteria was revised as needed. This has been further explained in the text to provide clarity. | Page 14, Data analysis, line 364 (Revised manuscript with track changes): “The research applied a narrative inquiry approach in data collection and analysis, which aims to capture respondents’ lived experiences and present a shared story which depicts themes and patterns based on the meaning and understanding that individuals give them (32). Therefore, data are presented descriptively to depict the patterns in these shared stories in terms of their narrative structure and content.  Data were coded using combination of a priori and inductive coding. An initial coding structure was developed using an adapted chrono-ecological framework (33,34), which examined the factors that influenced adolescent’s recruitment and reintegration at different levels of the ecological model (institutional, societal, community, interpersonal, and individual) over time (pre-conflict, conflict, and post-conflict). Using grounded theory, new codes were then added based on emergent themes and patterns in participants’ responses for each of these levels and time points within the chrono-ecological model. Detailed coding trees were then drafted with the combined deductive and inductive codes for each concept explored in the data and for each type of respondent, and a codebook was developed based on the coding tree.”  Regarding reliability of coding, see Page 14, Data analysis, Line 381 (Revised manuscript with track changes): “Data were coded by two members of the research team, with double-coding conducted on a random 5% of the articles. After an initial round of coding, the study team met to review the grounded codes and discuss and resolve any discrepancies and coding criteria was adjusted.” |
| 241 Analysis was completed in two phases: 1) first analyzing data from each country separately, then  242 2) synthesizing findings and identifying common themes and distinct differences between each country.  243 During the first phase of analysis, separate validation workshops were held with the CAR and DRC  244 programmatic teams (one for each country) including staff, caseworkers, and research team members  245 from all research sites. During the workshops, the research team led discussions on the codebook, initial  246 findings from that country, and points of clarification, and obtained crucial feedback for  247 contextualization.  **Comments:**  The procedures explained in Line 214 to 247 should have been briefly mentioned in research methodological approach to anticipate questions that the readers would ask about the different stages of analysis. | See the comment above regarding additional detail provided on the data analysis, analytical framework, and coding process, which have been added to the Methods sub-section on Data analysis (page 15) | See revisions detailed above. |
| *233 Transcripts and photographs of the life-lines were coded and analyzed using the qualitative*  *234 analysis software Dedoose. Data were analyzed using combination of a priori and inductive coding.*  *243 During the first phase of analysis, separate validation workshops were held with the CAR and DRC*  *244 programmatic teams (one for each country) including staff, caseworkers, and research team members*  *245 from all research sites.*  **Comments:**   - staff, caseworkers, and research team members should have been included in the sampling methods and their roles; this should be part of the research methodological approach. - Furthermore, the lack of research design statement makes it difficult to understand and put together the various roles played by research team, research assistants, staff, caseworkers, caregivers and child and adolescents involved with armed groups and forces.   The authors should address the preoccupation in the research method | Thank you for these comments. They have been addressed in the responses above and more detail has been added on the study design, sampling procedures, and research team make-up as suggested. | See revisions mentioned above. |
