# Supplementary material for: Drivers of ‘voluntary’ recruitment and challenges for families with adolescents engaged with armed groups: Qualitative insights from Central African Republic and Democratic Republic of the Congo
Source: PLOS Glob Public Health. 2023 May 24;3(5):e0001265. doi: 10.1371/journal.pgph.0001265 (PMC10208483; doi:10.1371/journal.pgph.0001265)
Supplement: Attachment — Submitted filename: Response to Reviewers.docx [file pgph.0001265.s004.docx]

10 March 2023

On behalf of the authorship team, we are pleased to resubmit the manuscript "Drivers of ‘voluntary’ recruitment and challenges for families with adolescents engaged with armed groups: qualitative insights from Central African Republic and Democratic Republic of Congo" with revisions. The authorship team thanks the reviewers for their thoughtful and helpful comments which have strengthened the paper. Each of the editorial and reviewer comments are addressed point-by-point in the table below, and revisions have been documented in track changes in the appropriate version.

We look forward to receiving your response to our revision.

Point-by-Point Reviewer Response

| **Comment** | **Response** | **Manuscript revisions** |
| --- | --- | --- |
| *Editorial feedback* | | |
| We appreciate the authors' careful attention to the comments. The manuscript is much improved, and the requested revisions are relatively minor clarifications. | Thank you for your thoughtful comments and attention to detail. The rounds of revision have greatly improved our manuscript. |  |
| Specifically, please address the comments related to the qualitative analysis, and clarify how grounded theory was employed. | These comments have been addressed in the manuscript and responded to below. |  |
| For Reviewer 4's comments concerning how to describe the locations and programs involved in the project, I defer to the authors' own IRB and advisory group requirements, given the sensitivity of the data and the need to protect participants and communities. I do agree that the "[REDACTED]" reads a little awkwardly, and simply referring to generic child protection programs (or another general description) would be sufficient. I also think that the definitions regarding adolescents and young adults are clearer, but check the manuscript to ensure the terminology is consistent throughout. | Thank you for this comment. In most places where we use “[REDACTED]”, this was for the purpose of retaining anonymity during the peer review process. We have reviewed each of these cases and referred to more generic child protection programs in some places and re-inserted the locations and programs involved, where appropriate.  We have also reviewed the terminology for adolescents and young adults and throughout the manuscript to ensure consistent terminology and provided definitions for these in the introduction of the manuscript. |  |
| Journal Requirements:  1. Please review your reference list to ensure that it is complete and correct. If you have cited papers that have been retracted, please include the rationale for doing so in the manuscript text, or remove these references and replace them with relevant current references. Any changes to the reference list should be mentioned in the rebuttal letter that accompanies your revised manuscript. If you need to cite a retracted article, indicate the article’s retracted status in the References list and also include a citation and full reference for the retraction notice. | We have added two references to the reference list but have not removed or retracted any articles. The additional references are noted here an mentioned in relation to the relevant revision below. | References added to reference list:  6. Erikson EH. Identity, youth and crisis. New York: Norton; 1968.  7. Sawyer SM, Azzopardi PS, Wickremarathne D, Patton GC. The age of adolescence. Lancet Child Adolesc Health. 2018;2(3):223–8.  15. Betancourt TS, Khan KT. The mental health of children affected by armed conflict: Protective processes and pathways to resilience. Int Rev Psychiatry Abingdon Engl. 2008 Jun;20(3):317–28.  16. Kohrt BA, Jordans MJD, Tol WA, Perera E, Karki R, Koirala S, et al. Social Ecology of Child Soldiers: Child, Family, and Community Determinants of Mental Health, Psychosocial Wellbeing, and Reintegration in Nepal. Transcult Psychiatry. 2010 Nov;47(5):10.1177/1363461510381290.  17. Cummings EM, Merrilees CE, Taylor LK, Mondi CF. Developmental and social–ecological perspectives on children, political violence, and armed conflict. Dev Psychopathol. 2017 Feb;29(1):1–10.  21. Poretti M. Preventing Children from Joining Armed Groups. Refug Surv Q. 2008 Dec 1;27(4):123–41.  References moved up within reference list:  32. Esposito J, Evans-Winters VE. Introduction to intersectional qualitative research. First Edition. Los Angeles: SAGE Publications, Inc; 2022. 206 p.  36. Bronfenbrenner U. The ecology of human development: Experiments by nature and design. Harvard university press; 1979.  37. Bronfenbrenner U, Morris PA. The Bioecological Model of Human Development. In: Handbook of child psychology: Theoretical models of human development, Vol 1, 6th ed. Hoboken, NJ, US: John Wiley & Sons Inc; 2006. p. 793–828. |
| 2. Please amend your detailed Financial Disclosure statement. This is published with the article. It must therefore be completed in full sentences and contain the exact wording you wish to be published.   a. State the initials, alongside each funding source, of each author to receive each grant. b. State what role the funders took in the study. If the funders had no role in your study, please state: “The funders had no role in study design, data collection and analysis, decision to publish, or preparation of the manuscript.” c. If any authors received a salary from any of your funders, please state which authors and which funders.  If you did not receive any funding for this study, please simply state: “The authors received no specific funding for this work.” | We have reviewed our Financial Disclosure statement and confirm it is correct and meets the necessary requirements. |  |
| 3. We have noticed that you have a list of Supporting Information legends in your manuscript. However, there are no corresponding files uploaded to the submission. Please upload them as separate files with the item type 'Supporting Information'. | We sincerely apologize for missing the supplemental information in the last manuscript. We have uploaded the corresponding files with this resubmission. |  |
| *Reviewer 1* | | |
| Publication requirements are met and detailed explanations regarding data availability have been provided by the authors. |  |  |
| First, I appreciate the authors' diligence in responding to the first round of reviewer comments. The responses were thoughtful and collegial, and the edits improved clarity of the paper. | Thank you for your thoughtful and detailed feedback and suggestions. We feel that they have enabled us to improve the manuscript. |  |
| I still have some concerns with the ways that the qualitative paradigm, methodology, and analytical methods are described. After reading the manuscript, appendices, and point-by-point comments, I feel as if the methodology is likely sound but is not being communicated in a way that shows the paradigmatic and methodological underpinnings. The use of in-depth interviews and the narrative lifeline elicitation technique is clear. However, narrative inquiry (a methodology) is mentioned in the analysis section. It is noted that the methodology guided "data collection and analysis". As a result, it should be placed earlier in the methods section so that the influence of narrative inquiry on the methods and data collection tools is clearer. | Thank you for this comment. We have moved the mention of narrative inquiry to earlier in the Methods section and have added more detail about how this methodology was utilized for both data collection and analysis. The use of in-depth interviews and the narrative lifeline technique—as well as the approach to analysis and presentation of the results—were informed by narrative inquiry with the objective of depicting the shared story of adolescents and families living in conflict areas with armed recruitment.  We also moved the Data analysis subsection up to be directly after the Data collection subsection to allow for continuity in description of the influence of narrative inquiry on the methods, and put the subsection on Ethical and safety considerations last. | Page 11, Methods, Data collection, line 247: “The research applied a narrative inquiry approach in data collection and analysis, which aims to capture respondents’ lived experiences and present a shared story which depicts themes and patterns based on the meaning and understanding that individuals give them (32). This methodology was employed to intentionally focus on the stories of conflict-affected families and their strategies of coping and forms of resistance told in their own words.”  References moved up within reference list:  32. Esposito J, Evans-Winters VE. Introduction to intersectional qualitative research. First Edition. Los Angeles: SAGE Publications, Inc; 2022. 206 p.  36. Bronfenbrenner U. The ecology of human development: Experiments by nature and design. Harvard university press; 1979.  37. Bronfenbrenner U, Morris PA. The Bioecological Model of Human Development. In: Handbook of child psychology: Theoretical models of human development, Vol 1, 6th ed. Hoboken, NJ, US: John Wiley & Sons Inc; 2006. p. 793–828.  Page 11-12, Methods, Data analysis: “Data were analyzed and presented descriptively utilizing narrative inquiry to depict the patterns in the shared stories of children and families experiencing armed recruitment in terms of their narrative structure and content.” |
| A second concern is related to the brief mention of grounded theory related to coding. Grounded theory encompasses much more than just the use of inductive coding, though that appears to be what is described by the authors here. I recommend interrogating the use of grounded theory or simply developed inductive codes. In its current form, I do not see the other elements of GT in the methodological framework. If such usual hallmarks were present in the study design, I recommend making these clearer. If they were not, it may be more accurate to state that inductive codes were used, without suggesting the use of GT. | We undertook significant work to develop and test the data collection tools and develop and refine the codebook and analytical framework in collaboration with key stakeholders and the study communities and programmatic teams in each research site. However, we agree that the analytic approach is more akin to inductive coding rather than a grounded theory approach, and have therefore removed reference to this throughout and focused on the inductive coding within the subsection on data analysis. | Pages 12-13, Methods, Data analysis (lines 289 and 293): Reference to “grounded theory” was removed and replaced with “inductive coding”. |
| Consistent with the SRQR reporting guidelines, consider a title that communicates the qualitative nature of the data. | We have revised the title to better convey the qualitative nature of the data. | Title: “Drivers of ‘voluntary’ recruitment and challenges for families with adolescents engaged with armed groups: qualitative insights from Central African Republic and Democratic Republic of the Congo” |
| *Reviewer 4* | | |
| Thank you for studying this important subject and carefully conduct research with such a vulnerable population group.  Here a few general comments and then others below by sections | Thank you for taking the time to review our manuscript and provide detailed comments. They have helped us to improve our paper. |  |
| 1. Whether to present the findings as a (new?) framework - I work in public health in conflict affected settings, but I am not familiar with the literature around voluntary child recruitment; it is therefore unclear to me if the authors use an existing framework or present a new one (which would justify the title). The four levels seem to be drawn and adapted from existing socio ecological frameworks. If the time component is the novelty, I would suggest to further describe and justify it. As it is now, it is mentioned once (line 306) but the rational why it should consider is not explained, i.e. why do you argue that factors evolve over time of the conflict? - It is therefore necessary to clarify whether you defined a new framework & what the novelty is, or whether you frame your results adapting/ using framework XY. - Regarding the time component, given that the insecurity situation continues in the two sites, I find it confusing to call the third phase “Post conflict”. I wonder whether these should be rather defined as “pre-recruitment”, “during recruitment”, “after involvement” (as you did in the pre-print version of the paper available online). | The model that we present serves to visually represent the findings using an adapted chrono-socioecological framework. This model builds on existing socioecological models of children and families in conflict settings but is adapted to proportionally represent the push and pull factors for engagement with armed groups as it was reported by adolescent and caregiver respondents. We do not intend to propose a new framework but rather present our empirical findings using this chrono-socioecological framing.  We have added to the introduction additional context on the existing socioecological models upon which this work builds, and clarified this further in the discussion where we present our graphical model of the results. We have also revised the title to emphasize the qualitative methodology and to minimize confusion over a new model. We have also revised the labels for the time component of the figure to refer specifically to periods of engagement with armed groups to align with your suggestion. | Page 5, Introduction, Socioecological theories on the experience of children and families in conflict, lines 96-105: “Existing socioecological theories (15-17) on the effect of conflict on child mental health and psychosocial wellbeing depict how risk and protective factors at different socioecological levels interact to drive recruitment into armed groups. These models emphasize the importance of child development and how risk factors introduced by conflict can directly drive poor developmental outcomes (e.g., traumatic war-related experiences, displacement, abduction, and community violence or affiliations, etc.) or indirectly exacerbate non-conflict risk factors (e.g. poverty and socioeconomic status, breakdown in social, economic, and service structures, and suppressed civil society, etc.) which in turn negatively affect risk factors at the interpersonal and individual levels. In contrast, protective factors at different socioecological levels could contribute to the strength and coping of young people living in conflict (15).”  New references added:  15. Betancourt TS, Khan KT. The mental health of children affected by armed conflict: Protective processes and pathways to resilience. Int Rev Psychiatry Abingdon Engl. 2008 Jun;20(3):317–28.  16. Kohrt BA, Jordans MJD, Tol WA, Perera E, Karki R, Koirala S, et al. Social Ecology of Child Soldiers: Child, Family, and Community Determinants of Mental Health, Psychosocial Wellbeing, and Reintegration in Nepal. Transcult Psychiatry. 2010 Nov;47(5):10.1177/1363461510381290.  17. Cummings EM, Merrilees CE, Taylor LK, Mondi CF. Developmental and social–ecological perspectives on children, political violence, and armed conflict. Dev Psychopathol. 2017 Feb;29(1):1–10.  21. Poretti M. Preventing Children from Joining Armed Groups. Refug Surv Q. 2008 Dec 1;27(4):123–41.  Page 29, Discussion, lines 753-762: “Building on existing socioecological models of children and families in conflict settings, this adapted model shows the drivers of recruitment from the perspectives of both the adolescent respondents and their caregivers at different levels of the chrono-socioecological framework. The model separates out the socioecological levels, with the size of each level denoting its relative importance as determined by occurrence in the transcripts. The time component is represented to emphasize the cyclical nature of conflict and recruitment in these settings and how these factors fluctuate throughout these periods of an adolescent’s experience. The separate depiction of adolescents and caregivers is not meant to contrast their perspectives but rather to draw out the central factors that both sets of respondents perceive to influence children to engage with armed groups, and to understand the overlapping needs of families living in conflict settings that affect household dynamics and erode the protective family environment.”  Page 30, Discussion: Fig 1 has been revised. |
| 2. The issue of using “children” or “adolescents” - Reviewer 3 (page 106) had previous raised the concern about the use of the words “Children” and “adolescents” (almost) interchangeably. There are still instances where only one of the two is used, and it is unclear if the other group is not included. It may be less confusing to use only one, or to clearly define the two groups at the beginning and then use each word appropriately. I would suggest in any case to add the definition of your study population from the introduction. As there is no legal definition of adolescents, you may want to stick to children. Or as respondents were finally aged 14 and above you may decide to use only adolescents. o For example, page 7, first sentence of Methods: “In depth qualitative interviews were conducted with adolescents and caregivers” (so no children?) - Also, it is confusing why respondents 18 to 20 were included, given that they are no longer (legally) children. You do state that they were asked about their experience when recruited as adolescents under the age of 18, but it may be good to know how many of the respondents belonged to each age group. | Within child development and psychosocial literature, adolescence is used to refer to a transitional developmental period between childhood and adulthood. Definitions of adolescents vary but generally comprise individuals from ages 10 to 24 (see Sawyer et al., 2018) to include children and young people of a specific social and cognitive developmental stage, rather than a specific age bracket (see Erikson, 1968). The terms “children” and “adolescents” are therefore not mutually exclusive.  For the purposes of this paper, the term “children” refers broadly to any person up to the age of 18. The majority of literature, evidence, and policy on child recruitment pertains to children under the age of 18, which has legal implications related to the age of majority. Adolescents are a group that includes older children. We use the term “adolescents” throughout the paper to refer to a specific subgroup of older children and young people within this developmental period, whereas the term “children” is used more broadly to refer to any age group of children from birth up to 18 years. These definitions have been added to the paper’s Introduction. Where children is used, the broader definition is intentional. Where adolescent is used, reference to this specific developmental period is intentional.  As described in the Methods, the study sample included adolescents ages 8 to 20 to capture the entirety of this developmental period across contexts. Older adolescents ages 18 to 20 were included in the sample so as to capture the experiences of these individuals when they were recruited as children, but also because young people ages 18 to 20 are still within this developmental stage of adolescence and experience the push and pull factors and the same negative consequences.  We have reviewed the paper to ensure that “children” and “adolescent” are used where appropriate and relevant. | Pages 3-4, Introduction, ‘Voluntary’ recruitment of children to armed groups, lines 69-74: “Within child development theory, adolescence is the transitional psychosocial stage between childhood and adulthood (6) which can occur from around age 10 to age 24 (7). For the purpose of this study, we use the term “adolescents” to refer to a specific subgroup of older children (aged 8 years and above) within this transitional period, whereas the term “children” is used more broadly to refer to any age group of children from birth up to 18 years.”  References added for this revision:  6. Erikson EH. Identity, youth and crisis. New York: Norton; 1968.  7. Sawyer SM, Azzopardi PS, Wickremarathne D, Patton GC. The age of adolescence. Lancet Child Adolesc Health. 2018;2(3):223–8.  Page 8, Methods, Study participants, lines 190-193: “Older adolescents ages 18-20 were included so as to gather information about their experiences when recruited as adolescents under the age of 18, and because this age group includes young people within the transitional developmental stage of adolescence.” |
| 3. Ensuring confidentiality and protection of the study population. While I recognize the importance of this, using the word “REDACTED” is confusing (when reading) and I think not sufficient: - As the majority of the authors work for IRC, the reader will likely quickly assume that IRC is implementing a child protection program and that participants come from this program. Looking up the “Safe Healing and Learning Spaces” (that you mention on page 11) leads you to the IRC website. - I would suggest to state that neither precise locations nor names of the child protection organizations working in each country will be made for security and confidentiality reasons. You can then remove the “redacted” and just refer to “the child protection organization”. - I would suggest to keep the locations as Ouham Pende (as it is a prefecture, I assume it is big enough), and Nord Kivu (without stating Nyriagongo like on page 11). - From a methodological point of view and for the sake of transparency I think it is important to state that IRC implements child protection projects in the areas to exclude (or not) possible recruitment biases. - I leave it to the editor to decide how to best deal with this. | Thank you for your suggestions for maintaining confidentiality. We have incorporated these throughout the manuscript.  The use of “REDACTED” was for the purpose of retaining anonymity during the review process but we realize this is not necessary given that author information is shared simultaneously. We have reviewed each of these cases and referred to more generic child protection programs in some places and re-inserted the organization name, where appropriate for transparency and reporting purposes. | Revisions have been made throughout the manuscript. For example:  Page 12, Methods, Data collection, line 270: “In-depth interviews were conducted with adolescents and caregivers across three sites in Ouham-Pendé, CAR in March 2020 and five sites in North Kivu, DRC in October 2020.”  Page 12, Methods, Data collection, line 274: “Interviews were conducted either in a designated programmatic space or in a separate safe space of the respondent’s choosing.” |
| Methods - Inclusion criteria: o Line 174: only “formerly” is stated while Line 176 “formerly or currently involved” ◊ please confirm if it is only formerly or also currently. - Line 177: Languages spoken: Swahili and Kinyarwanda are mentioned, which I assume apply to DRC. Need to add Sango for CAR to align with line 253. | Thank you for these revisions, we have incorporated them into the manuscript. | Page 8, Methods, Study participants, lines 180-182: “To better understand the experiences of adolescents and their families living in conflict settings, in-depth interviews were conducted with adolescents currently or formerly engaged in armed groups or at risk of joining and their caregivers.”  Page 8, Methods, Study participants, lines 182-184: “Inclusion criteria for the study were adolescents aged 8-20 years who were either currently or formerly involved in armed groups or were determined to be at risk of joining and speakers of Swahili, Kinyarwanda, Sango, or French languages.” |
| - Sample size: o Could you clarify if the calculations were made for the children (only) or for both children and caregivers? o It sounds as though you had to modify the sample size calculation (i.e., I assume reducing the sample size?) from your initial approach as you had challenges in recruiting girls and at risk children. o The second (revised approach) proposes to sample 10% of the verified cases. You state there are ♣ 442 cases in DRC ◊ target sample size 45 ♣ 75 cases in CAR ◊ however the target sample size is 35 and is based on “310 cases of grave violations”. It seems the word case is used for children in DRC and for events in CAR? could you provide a definition of a case? o How did you decide to interview 39 caregivers? | We confirm that calculations were made for the children only. The sample size for caregivers was based on the caseload for each country, for which we aimed to interview 20 for each country (40 total), 50% men and women. We have specified in the manuscript that the sample size calculation was specific to the adolescent sample.  Due to challenges with recruiting adolescent participants using the first approach applied in CAR, the sample size calculation for adolescents was revised and increased in the second approach that was applied in the DRC. This was based on an approximation of reported child recruitment or conflict-related grave violations against children, depending on what data was available for each country. We recruited additional respondents in CAR after difficulty recruiting adolescent girls and at-risk respondents to reach saturation and over-sampled these populations in the DRC to account for these challenges.  We have revised the language and description to clarify the sampling approach. | Page 90, Methods, Study participants, lines 216-219: “However, after some initial challenges identifying adolescent girl respondents and at-risk respondents during data collection in CAR that inhibited reaching data saturation, the sampling approach was altered and increased based on learning from this process.”  Pages 9-10, Methods, Study participants, lines 219-231: “This involved determining the target sample size by calculating approximately 10% of the verified cases of child recruitment in each region. Verified cases are those cases that are verified by the UN as part of their monitoring and reporting mechanism (MRM), through which teams on the ground collect information and respond to grave violations against children. According to the UN General Assembly Security Council resolution 2427 (3), 442 children were recruited by armed groups in North Kivu, DRC (target N=45 adolescents). While the number of children recruited by armed groups was not reported by prefecture in CAR, there were 310 cases of conflict-related grave violations in Ouham-Pendé (target N=35 adolescents) in 2018. As a result, additional interviews were conducted in CAR and adolescent girls were oversampled in DRC to ensure adequate representation of the experiences of girls in the study results. This enabled a more strategic sampling approach and the saturation of themes across research sites in both countries. Caregivers were sampled based on the caseload for each country, with a target sample of 20 caregivers and equal representation of men and women respondents.” |
| - Table 1: “out of armed groups” does it mean that none of the respondents were currently involved? | “Out of armed groups” is used to refer to those adolescents who were currently or formerly engaged with armed groups but were currently living at home. This term is used by the local community and was therefore used to categorize the respondents for this study. | Page 11, Methods, Study participants, line 234: “Adolescent respondents who were currently or formerly engaged with armed groups but were currently living in their households are categorized as “out of armed groups”. |
| - Ethical consideration: o Suggest moving lines 160 to 171 to this section. | Thank you for this suggestion, we have moved these lines to the Ethical and safety considerations subsection. | Page 13, Methods, Ethical and safety considerations, line 316: “The research protocol for this study was reviewed and approved by the International Rescue Committee institutional review board…” |
| - Coding: o Line 314 (manuscript) vs response to reviewer 1 (page 92): it seems misleading to state that data were coded by two members, when you answered that it was coded by one member except for the initial 5% used to ensure coordination of coding. I would therefore say that “two members coded 5% of the transcripts to harmonize approaches, after which one team member coded all the remaining transcripts”. | We would like to clarify that transcripts were indeed divided and coded between two members of the research team. Only 5% of transcripts were double-coded and a harmonization meeting was held after this double-coding to discuss any differences between these two coders. However, both team members continued with coding the remaining transcripts after harmonization.  This sentence has been revised for clarity. | Page 13, Methods, Data analysis, lines 296-299: “Two members of the research team conducted the coding of transcripts, with double-coding conducted on a random 5% of the articles.” |
| Results - Line 349: “also” ◊ this is the first factor that is reported, better to remove “also”. | This revision has been incorporated into the text. | Page 16, Results, line 381: “Economic drivers were reported by boys and girls in CAR and DRC…” |
| - Line 358: remove CAR, as data were collected in March 2020 in CAR, ie. very early in the COVID pandemic. | The finding that the conflict situation was exacerbated by COVID-19 is evident from the research conducted and validation workshops held with programmatic staff and other stakeholders. We have therefore determined not to remove the reference to CAR in this sentence. |  |
| - To be formatted as quotes: o Lines 385 to 387 o Lines 479 - 481 o Lines 518 - 520 o Lines 524 – 526 o Lines 558 – 560 o Lines 576 – 577 o Lines 609 – 611 o Lines 612 - 613 o Lines 697 - 699 | We have followed Vancouver Style formatting for quotations. Short quotations (less than 40 words) are presented in-text with quotation marks, whereas long quotations are laid out in block quotes using a separate paragraph of text and indented from the left margin.  We have reviewed the quotes that you highlighted in your comment and ensured that any quotes over 40 words are formatted as block quotes. For those few quotes that are shorter, we have left them in-text. |  |
| - Lines 497-501: the quote does not support the statement made on lines 497, rather says the opposite. | The last line in the quote is about the three years that she stayed with the armed group because her life at home was also difficult. The preceding sentence providing context has been revised for more clarity. | Page 21, Results, Conflict, line 530: “One 18-year-old girl in the DRC explained that she stayed longer with the group because she perceived her situation at home to be the same:” |
| Discussion - Fig 1: you should add in the methodology how you defined and counted the occurrence of certain factors, i.e. “the numeric findings” behind this figure, and how it is reflected in the size of the circles. Is this something that was done in the software you used? | The occurrence of certain factors and their perceived importance were determined based on the frequency of factors mentioned by adolescent and caregiver respondents. Given that these were qualitative findings from in-depth interview transcripts, the numeric frequencies have not been reported. However, they have been graphically represented in the visual framework in Fig. 1, which was developed manually and validated by stakeholders from both countries. More detail on the graphical representation of the findings has been added to the Methods subsection on Data analysis. | Page 13, Methods, Data analysis, lines 306-312: “The research team then synthesized findings from both countries, and re-presented the findings and the resulting visual framework to the programmatic teams in both countries, as well as the external technical advisory group for final validation. Perceived importance of risk and protective factors for recruitment and barriers and facilitators of reintegration were determined based on the frequency of factors mentioned by adolescent and caregiver respondents. These have been graphically represented in the visual framework in Fig 1, with the size of each level denoting its frequency of occurrence in the transcripts relative to other levels.” |
| - The discussion is long and includes some repetitions from the results. As the result section is long, I would suggest cutting down the discussion and keep only key points. | We acknowledge the length of the discussion but have decided to retain its content. The discussion is currently structured based on in-depth feedback and requests for additional context and explanation that were added in response to past stages of review. It provides a summary of our key findings from the results and supports or contrasts our qualitative findings with evidence from other empirical research, which is essential for contributing generalizable findings to the evidence base. |  |
